# Identification of SGK1 as a candidate regulator associated with myeloid cell senescence in ME/CFS: An integrative transcriptomic and experimental study

**DOI:** 10.1371/journal.pone.0359082

**Published:** 2026-10-16

**Authors:** Yan Jin, Rui Qin, Wan Li, Jin Liu, Xinghua Chen

**Affiliations:** 1 The First Affiliated Hospital of Guangzhou University of Chinese Medicine, Guangzhou, China; 2 Guangzhou University of Chinese Medicine, Guangzhou, China; Army Medical University, CHINA

## Abstract

**Background:**

Myalgic encephalomyelitis/chronic fatigue syndrome (ME/CFS) is a complex illness lacking objective diagnostic biomarkers and clear pathophysiological understanding. Patients exhibit immune dysregulation and chronic inflammation reminiscent of aging phenotypes, yet the role of myeloid cell senescence in this disease remains unexplored.

**Methods:**

A stepwise integrative transcriptomic and computational strategy was employed. Differentially expressed genes were identified from the bulk transcriptomic dataset GSE14577. Weighted gene co-expression network analysis was performed to identify key modules correlated with the ME/CFS phenotype. Candidate genes were obtained by intersecting these modules with aging-related genes from the GenAge database. Core hub genes were further selected using LASSO regression and random forest machine learning algorithms. Cell type-specific expression was mapped using single-cell RNA sequencing data GSE214283, and functional validation was conducted via siRNA knockdown in a THP-1 cell senescence model.

**Results:**

Integrative analysis identified HMGCR and SGK1 as core hub genes. Functional enrichment analysis revealed that candidate genes were enriched in pathways related to cellular senescence and inflammation. Single-cell transcriptomics analysis suggested that SGK1 expression was enriched in myeloid cell populations, including monocytes and neutrophils, in individuals with ME/CFS, whereas HMGCR showed a broader expression pattern. Single-gene GSEA indicated that SGK1 was associated with autophagy and lysosomal function. In vitro experiments showed that SGK1 expression was significantly upregulated in D-galactose-induced senescent THP-1 cells, and SGK1 knockdown markedly reduced the mRNA expression levels of senescence markers p16, p21, and p53.

**Conclusion:**

This study identifies SGK1 as a candidate molecule associated with myeloid cell senescence-related alterations in ME/CFS. Integrated transcriptomic, single-cell, and experimental analyses suggest a potential link between SGK1 expression and aging-associated immune alterations. However, further validation in primary ME/CFS-derived immune cells and clinical cohorts is required to determine the biological significance and clinical relevance of SGK1.

## 1. Introduction

Myalgic encephalomyelitis/chronic fatigue syndrome (ME/CFS) is a complex illness characterized by persistent or relapsing fatigue, post-exertional malaise, cognitive impairment, and unrefreshing sleep, affecting approximately 0.4% to 1.0% of the global population [1]. Diagnosis currently relies on clinical criteria without objective biomarkers, leading to diagnostic delays and frequent misdiagnosis. Available therapeutic options remain limited and controversial; the 2021 update of the NICE guideline was criticized for lacking robust evidence, underscoring the need for high-quality research to establish a mechanistic basis for this condition [2]. Elucidating the pathophysiological mechanisms of ME/CFS and identifying key molecular targets are therefore prerequisites for developing objective diagnostic tools and effective therapeutic strategies.

Aging is a complex process driven by multiple molecular hallmarks, systematically conceptualized as twelve hallmarks of aging encompassing genomic instability, mitochondrial dysfunction, cellular senescence, and chronic inflammation [3]. Patients with ME/CFS exhibit clinical and molecular features consistent with several of these hallmarks, including chronic low-grade inflammation, mitochondrial dysfunction, and immune cell abnormalities [4]. Cardiovascular alterations such as reduced cardiac output and structural cardiac changes have been documented in ME/CFS, implicating energy metabolism disturbance as a core feature of the disease [5]. These observations have led to the hypothesis that ME/CFS may represent a state of accelerated biological aging, at least in certain physiological systems. Among the twelve hallmarks, cellular senescence has garnered particular attention for its role in driving inflammation and tissue dysfunction. The inducing mechanisms and core features of cellular senescence have been comprehensively delineated, including irreversible cell cycle arrest mediated by p53-p21 and p16-pRB pathways, increased lysosomal activity, and metabolic alterations [6]. Senescent cells exert paracrine effects through the senescence-associated secretory phenotype (SASP) [7], secreting pro-inflammatory cytokines, chemokines, and proteases that influence the surrounding microenvironment. The intricate regulatory networks of the SASP and its central role in inflammaging have been elucidated, with particular emphasis on immune cell senescence, especially macrophage and monocyte senescence, as a key driver of chronic inflammation [8]. Senescent myeloid cells exhibit impaired phagocytic capacity and an enhanced pro-inflammatory phenotype, affecting neighboring immune and tissue cells through SASP factor secretion [9]. Investigating the senescent state of myeloid cells in ME/CFS may therefore provide insights into immune dysregulation and chronic inflammatory mechanisms in this disease.

Recent advances have begun to unravel the complexity of ME/CFS. A recent artificial intelligence-driven multi-omics study integrated gut metagenomic, plasma metabolomic, and immune cell profiling data to construct a “microbiome-immune-metabolome” axis interaction map in ME/CFS, identifying key alterations such as activation of mucosal-associated invariant T cells and γδ T cells [10]. This work provided a systems-level perspective on the heterogeneity of ME/CFS. Concurrently, progress has been made in diagnostic biomarker discovery, with the first proposed blood test for ME/CFS based on single-cell Raman spectroscopy being reported [11]; however, the scientific community has expressed reservations regarding its limited sample size, potential reproducibility concerns, and lack of mechanistic clarity, emphasizing that advancing diagnostic technologies must be accompanied by deeper understanding of the underlying molecular mechanisms [12]. Despite these advances, the core molecular events and specific cell types driving the complex phenotypes in ME/CFS remain incompletely defined. In particular, the senescent status of myeloid cells and its regulatory factors in ME/CFS have not been investigated to date.

SGK1 (serum/glucocorticoid regulated kinase 1) is a stress-responsive serine/threonine kinase involved in inflammatory signaling and cellular stress responses. Previous studies have linked SGK1 to pathways such as NF-κB and mTOR signaling, which are also implicated in senescence-related processes. However, its relevance to aging-associated alterations in ME/CFS remains unknown [13,14]. A stepwise screening strategy was employed: differentially expressed genes were identified from the bulk transcriptomic dataset GSE14577; key modules correlated with the ME/CFS phenotype were identified through weighted gene co-expression network analysis; candidate genes were obtained by intersecting with the GenAge database of aging-related genes; core hub genes were selected using LASSO regression and random forest machine learning algorithms; cell type-specific expression was localized using single-cell RNA sequencing data GSE214283; and finally, candidate genes emerging from the integrative analysis were further investigated for their potential association with myeloid cell senescence-related alterations through single-cell characterization and in vitro experiments using a THP-1 cell senescence model and gene-specific knockdown approaches.

## 2. Materials and methods

### 2.1 Data acquisition and preprocessing

The bulk transcriptomic dataset GSE14577 was obtained from the Gene Expression Omnibus (GEO) database and used as the discovery cohort in this study. Among publicly available ME/CFS transcriptomic datasets, GSE14577 was selected as the discovery cohort because it provides clinically characterized PBMC transcriptomic profiles from post-infectious ME/CFS patients and matched healthy controls, with available raw expression data suitable for differential expression analysis and co-expression network construction. The well-defined clinical phenotype and availability of both patient and control samples allowed this dataset to serve as a hypothesis-generating discovery cohort for identifying aging-related and senescence-associated molecular signatures. According to the original study description, GSE14577 included PBMC samples from 8 male patients with well-characterized post-infectious ME/CFS and 7 age-matched male healthy controls. All patients underwent clinical and neurological evaluation, and alternative explanations for symptoms were excluded. Importantly, all patients fulfilled the 1994 Centers for Disease Control and Prevention (CDC) criteria (Fukuda criteria) for chronic fatigue syndrome diagnosis and had a documented history of viral or bacterial infection preceding persistent fatigue. The median age of patients was 36 years (range 18–54 years), and the median disease duration was 4.5 years. Female participants were not included in the original study to minimize potential confounding effects related to reproductive cycles and contraception. The dataset was therefore used as a discovery cohort for hypothesis generation and candidate molecular signature identification, rather than definitive biomarker validation, while recognizing limitations associated with the small sample size and sex-specific composition. The original study generating GSE14577 primarily investigated transcriptional alterations associated with post-viral fatigue by identifying differentially expressed genes between patients and healthy controls. In contrast, the present study applied an independent integrative analytical framework focusing on aging-related and senescence-associated molecular alterations. Specifically, we combined aging-related gene profiling, weighted gene co-expression network analysis (WGCNA), machine learning-based feature selection, single-cell transcriptomic characterization, and experimental investigation to identify and evaluate candidate molecular signatures associated with ME/CFS-related senescence.

Raw CEL files were processed using the Robust Multi-array Average (RMA) algorithm for background correction, quantile normalization, and probe summarization. For genes represented by multiple probes, the median expression value was assigned as the final expression level for that gene. Differential expression analysis was performed using the “limma” package in R. Linear models were constructed, and standard errors were moderated using an empirical Bayes method. Differentially expressed genes (DEGs) were identified based on the following thresholds: an adjusted P-value (False Discovery Rate, FDR) < 0.05 and an absolute log2 fold change (|log2(FC)|) > 0.5. A list of human aging-related genes (ARGs) was obtained from the human gene dataset of the GenAge database within the Human Aging Genomic Resources (HAGR; https://genomics.senescence.info/). Only human ARGs included in the GSE14577 dataset were retained for subsequent analyses. The GenAge model organism datasets were not included in the present analysis. Additionally, an independent PBMC RNA-seq dataset (GSE251872) was obtained from the GEO database for external evaluation of hub gene expression patterns. This dataset was used to evaluate SGK1 and HMGCR expression patterns in an independent ME/CFS cohort.

### 2.2 Weighted Gene Co-expression Network Analysis (WGCNA)

To identify gene modules highly correlated with the ME/CFS phenotype, a weighted gene co-expression network was constructed using the “WGCNA” package in R [15]. Sample clustering was performed to evaluate potential outliers before WGCNA analysis. One sample (GSM364560) showing marked deviation from the remaining samples in hierarchical clustering was identified as an outlier and excluded from subsequent network construction. Therefore, WGCNA was performed using the remaining 14 samples (8 ME/CFS samples and 6 healthy controls). This outlier exclusion step was applied only for co-expression network construction and was not applied to differential expression analysis. Importantly, exclusion of GSM364560 was restricted to WGCNA network construction and did not affect differential expression analysis or the initial identification of candidate genes. A pairwise Pearson correlation matrix was calculated for all genes, and a soft-thresholding power (β) was selected to achieve a scale-free topology (typically requiring a scale-free topology fit index R² > 0.85 and a mean connectivity close to zero). Based on the Topological Overlap Matrix (TOM), gene dissimilarity (1-TOM) was computed. Gene modules were identified by cutting the hierarchical clustering dendrogram using the dynamic tree cut algorithm (minimum module size = 30; merge cut height = 0.25). Each module was assigned a unique color identifier. Module eigengenes (MEs) were calculated for each module, and their correlations with the clinical trait (ME/CFS status) were assessed. Modules showing the highest absolute correlation coefficients with statistical significance (*P* < 0.05) (specifically, the Purple and Darkgreen modules) were selected as key modules, and all genes within these modules were extracted for further screening.

### 2.3 Candidate gene selection and functional enrichment analysis

To obtain core genes characterized by differential expression, co-expression module membership, and aging relevance, a multi-set intersection strategy was employed. The final candidate gene list was generated by taking the intersection of three sets: DEGs, genes from the key WGCNA modules (Purple and Darkgreen), and ARGs from the HAGR database. Gene Ontology (GO) and Kyoto Encyclopedia of Genes and Genomes (KEGG) pathway enrichment analyses were performed on this candidate list using the “clusterProfiler” package in R. GO analysis covered biological processes (BP), cellular components (CC), and molecular functions (MF). Enrichment significance was evaluated using the hypergeometric test, and P-values were adjusted for multiple testing using the Benjamini-Hochberg method. Terms and pathways with an FDR < 0.05 were considered statistically significant. Given the limited number of candidate genes, enrichment results that did not survive multiple-testing correction were considered exploratory and interpreted cautiously. Results were visualized using bubble plots and bar charts.

### 2.4 Machine learning-based hub gene selection

To further refine the candidate genes into a robust set of diagnostic hub genes, we employed two machine learning algorithms: Least Absolute Shrinkage and Selection Operator (LASSO) [16] regression and Random Forest (RF) [17]. First, a LASSO logistic regression model was constructed using the “glmnet” package. Ten-fold cross-validation was performed to plot the deviance against the log(λ) curve. Genes with non-zero coefficients at both the minimum lambda (lambda.min) and the one-standard-error lambda (lambda.1se) were retained as the LASSO feature set. Concurrently, a Random Forest classifier was built using the “randomForest” package with 1,000 trees (ntree = 1000). The importance of each gene was quantified by the Mean Decrease Gini index. Genes ranking high in importance were selected as the RF feature set. The intersection of genes selected by both LASSO and RF algorithms was defined as the final hub genes for this study.

### 2.5 Single-gene stratified gene set enrichment analysis

To explore biological pathways associated with the identified hub genes, single-gene stratified Gene Set Enrichment Analysis (GSEA) was performed. For each hub gene (HMGCR and SGK1), samples from the GSE14577 dataset were stratified into high- and low-expression groups according to the median expression level of the corresponding gene. Differentially expressed genes between the high- and low-expression groups were identified using the limma package and ranked according to their expression changes (S1A Fig, S1B Fig). Gene Set Enrichment Analysis was subsequently performed using Hallmark and KEGG gene sets from the Molecular Signatures Database (MSigDB). Enriched pathways associated with higher expression of HMGCR or SGK1 were visualized using enrichment plots and pathway ranking analyses.

### 2.6 Single-cell transcriptomic data processing and analysis

The single-cell RNA sequencing (scRNA-seq) datasets GSE214283 and GSE157007 were downloaded from the Gene Expression Omnibus (GEO) database. GSE214283 contains PBMC single-cell transcriptomic profiles from individuals with ME/CFS and healthy controls and was used to characterize the cell-type distribution and expression patterns of the candidate hub genes identified from bulk transcriptomic analysis. GSE157007 was included as an aging reference cohort to explore whether similar expression patterns were observed in age-associated immune alterations.

Because the objective of the single-cell analysis was exploratory characterization of cell-type-specific expression patterns rather than population-level biomarker validation, a representative subset of samples was selected for integrated analysis. Specifically, three ME/CFS samples and three healthy control samples were randomly selected from GSE214283. In addition, three healthy control samples and three aging samples were selected from GSE157007. These 12 samples were integrated to compare SGK1 and HMGCR expression patterns across ME/CFS-associated and aging-associated immune states.

Data processing and analysis were conducted using the “Seurat” package in R. Quality control was performed by filtering out cells with mitochondrial gene content > 20%, unique molecular identifier (UMI) counts < 200 or > 2500, or fewer than 200 detected genes. Data were normalized using the LogNormalize method (scale factor = 10,000), and the top 2,000 highly variable genes (HVGs) were identified. Principal Component Analysis (PCA) was performed, and significant principal components were selected based on Elbow and JackStraw plots. Non-linear dimensionality reduction was achieved using Uniform Manifold Approximation and Projection (UMAP). Unsupervised clustering was performed using the Shared Nearest Neighbor (SNN) graph algorithm with a resolution parameter between 0.5 and 1.0. Cell types were annotated based on the expression of canonical marker genes. Differential expression of hub genes across disease states (Control, ME/CFS, Aging) and cell subpopulations was analyzed using the “FindMarkers” function (Wilcoxon rank-sum test) and visualized using UMAP feature plots and dot plots.

### 2.7 In vitro cell experiments

Cell Culture and Senescence Induction: The human monocytic leukemia cell line THP-1 was used as an in vitro model to simulate PBMC pathophysiology in ME/CFS and aging. Cells were cultured in RPMI-1640 medium supplemented with 10% fetal bovine serum (FBS), 1% penicillin-streptomycin, and 0.05 mM β-mercaptoethanol at 37°C in a 5% CO_2_ humidified incubator. To establish a cellular senescence model, THP-1 cells were divided into two groups: (1) Control group: treated with an equal volume of PBS; and (2) β-gal induction group: treated with D-galactose (20 mM) for 48–72 hours. Following treatment, total RNA was extracted from both groups. The mRNA expression levels of classic senescence markers (p21, p16, p53) and the hub gene (SGK1) were quantified using quantitative real-time PCR (qRT-PCR).

### 2.8 Gene knockdown and assessment of senescence markers

To investigate the regulatory role of the hub gene in cellular senescence, loss-of-function experiments were performed. THP-1 cells were transfected with either non-targeting scrambled siRNA (si-NC group) or specific siRNA targeting SGK1 (si-SGK1 group) using Lipofectamine 3000 according to the manufacturer’s protocol. After 24 hours of transfection, cells were harvested for total RNA extraction. The knockdown efficiency of SGK1 and the subsequent changes in mRNA expression levels of senescence markers (p21, p16, p53) were assessed by qRT-PCR. All qRT-PCR results were calculated using the 2 − ΔΔCt method, with GAPDH serving as the internal reference gene. All experiments were performed in independent triplicates.

### 2.9 Statistical analysis

All bioinformatic analyses were performed using R software (version 4.3.3) (S1 Code). Comparisons between two groups were conducted using the unpaired Student’s t-test (for normally distributed data with equal variance) or the Wilcoxon rank-sum test (for non-normally distributed data). Comparisons among multiple groups were performed using one-way analysis of variance (ANOVA) followed by Tukey’s post-hoc test, or the Kruskal-Wallis test followed by Dunn’s test, as appropriate. Correlations were assessed using Pearson or Spearman coefficients. All statistical tests were two-tailed, and an adjusted *P*-value < 0.05 was considered statistically significant. Data visualization was generated using R packages including “ggplot2”, “pheatmap”, “ComplexHeatmap”, “cowplot”, and “Seurat”. Experimental data are presented as mean ± standard deviation (SD). Statistical graphs for in vitro experiments were created using GraphPad Prism software.

## 3. Results

### 3.1 Study overview and identification of differentially expressed genes

The overall study design and analytical workflow are illustrated in Fig 1. To explore the molecular signatures of ME/CFS, we first analyzed the bulk transcriptomic dataset GSE14577. Following rigorous quality control and normalization using the Robust Multi-array Average (RMA) algorithm, differential expression analysis identified 98 DEGs between ME/CFS patients and healthy controls (criteria: |log2FC| > 0.5, FDR < 0.05). As depicted in the heatmap and volcano plot (Fig 2A, 2B, S1 Table), these DEGs exhibited distinct expression patterns, with a subset significantly upregulated and another downregulated in ME/CFS samples, forming the foundational dataset for subsequent analyses. Given that GSE14577 represents a small, male-only discovery cohort, the subsequent analyses were designed to identify candidate molecular associations rather than establish definitive population-level biomarkers. Unlike the original analysis of GSE14577, which primarily focused on identifying differentially expressed genes between ME/CFS patients and controls, the present study applied an integrative analytical framework incorporating aging-related gene profiling, co-expression network analysis, machine learning, single-cell transcriptomics, and experimental assessment to explore senescence-associated molecular signatures in ME/CFS.

**Fig 1:**
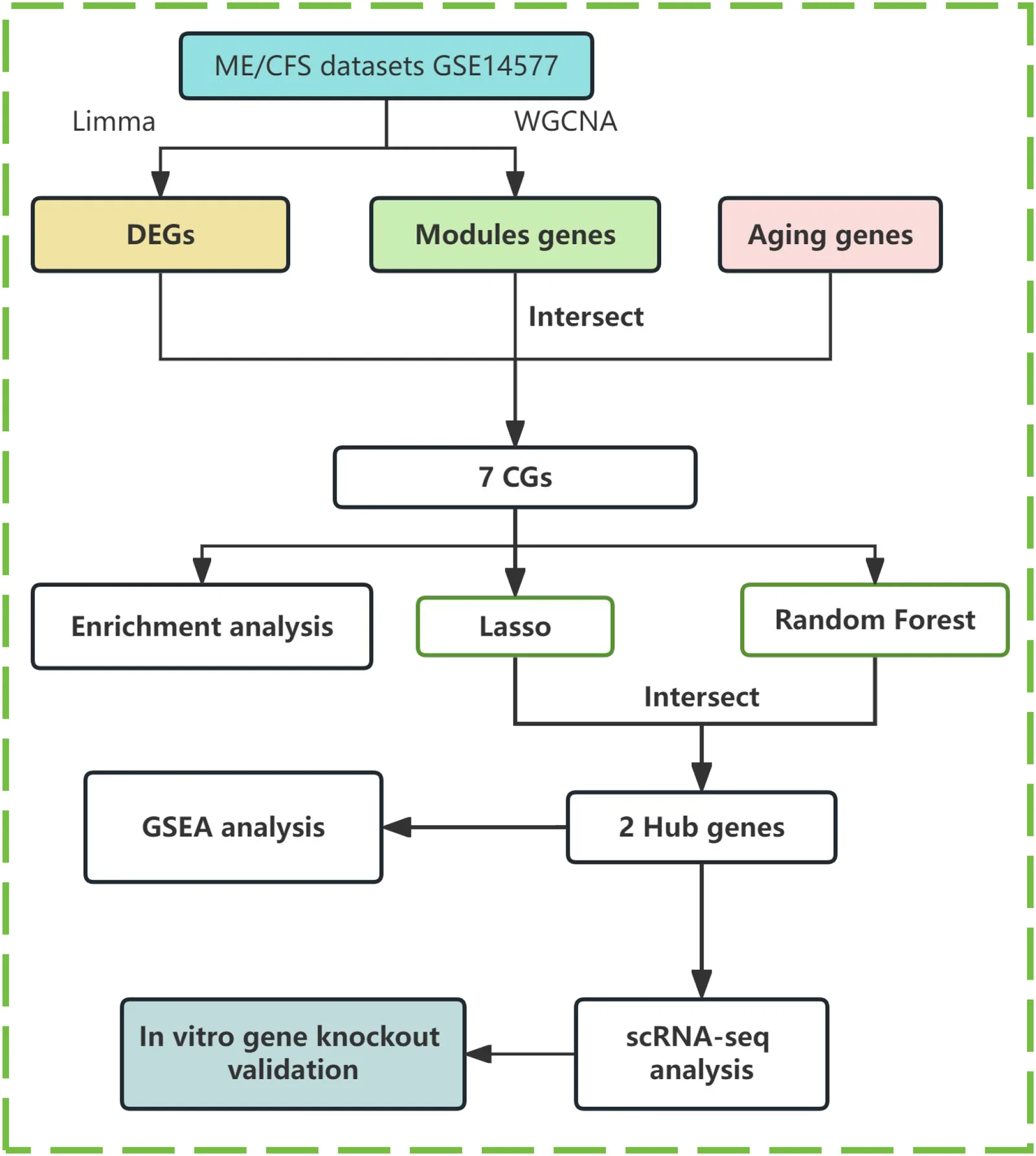
Flowchart of the study design and analytical workflow.

**Fig 2:**
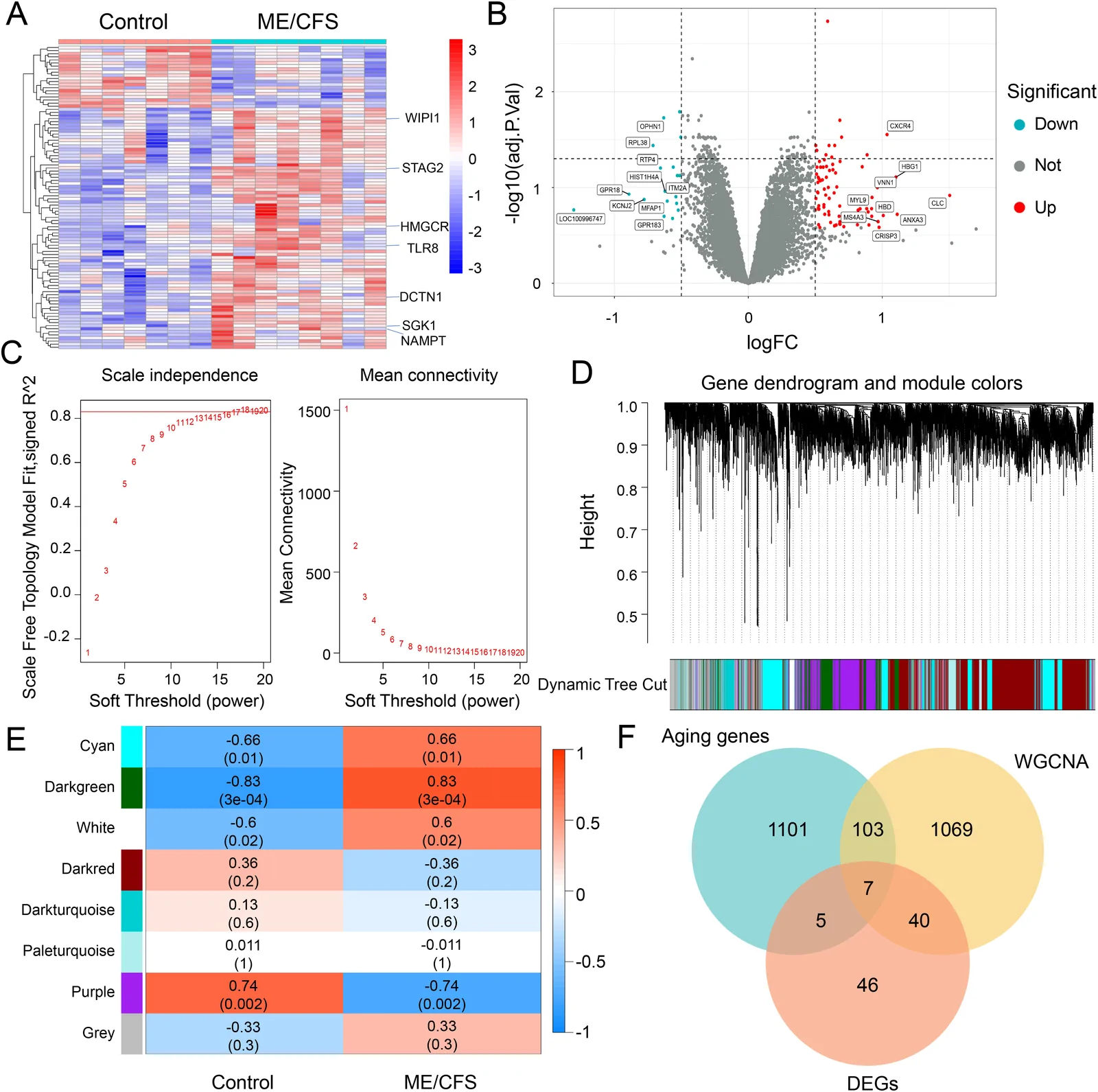
Identification of aging-related differentially expressed genes in ME/CFS. A: Heatmap showing the expression patterns of differentially expressed genes (DEGs) between Control and ME/CFS samples. Red indicates high expression, blue indicates low expression. B: Volcano plot illustrating DEGs. Red dots: up-regulated genes; Blue dots: down-regulated genes; Gray dots: non-significant genes (|logFC| > 0.5, adj.P.Val < 0.05). C: Determination of soft-thresholding power in WGCNA. Left: Scale-free topology fit index (R^2^); Right: Mean connectivity. D: Gene dendrogram and module colors. Each branch represents a gene, and modules are assigned distinct colors based on hierarchical clustering. E: Module-trait relationships heatmap. Correlation coefficients and P-values between gene modules (rows) and clinical traits (Control/ME/CFS, columns). F: Venn diagram showing the intersection of Aging genes, WGCNA module genes, and DEGs, yielding 7 candidate genes (CGs).

### 3.2 WGCNA reveals key gene modules highly correlated with ME/CFS phenotypes and candidate gene selection

To uncover co-expression networks tightly associated with the ME/CFS phenotype, we constructed a Weighted Gene Co-expression Network Analysis (WGCNA). After hierarchical clustering-based quality assessment, one outlier sample (GSM364560) was excluded from network construction. Therefore, WGCNA was performed using the remaining 14 samples (8 ME/CFS samples and 6 healthy controls). A soft-thresholding power of β = 18 was selected to achieve a scale-free topology fit index (R^2^) > 0.85 (Fig 2C). Hierarchical clustering based on the TOM identified 8 distinct gene modules (Fig 2D). Correlation analysis between module eigengenes (MEs) and clinical traits revealed that the Purple and Darkgreen modules showed the strongest associations with the ME/CFS phenotype (*P* < 0.05; Fig 2E). These modules displayed significant correlations with disease status, with one module showing a positive correlation and the other showing an inverse correlation with the ME/CFS phenotype. Consequently, these two modules were designated as key modules, encompassing 1,219 genes potentially associated with ME/CFS-related transcriptional alterations. To pinpoint core candidate genes integrating differential expression, module membership, and aging relevance, we employed a multi-set intersection strategy. By intersecting the 98 DEGs, the 1,219 genes from the key WGCNA modules, and human Aging-Related Genes (ARGs) from the GenAge (307 genes) and CellAge (279 genes) databases, we ultimately identified 7 candidate genes (CGs) potentially associated with ME/CFS-related aging and senescence signatures (Fig 2F).

### 3.3 Functional enrichment analysis of candidate genes

Subsequently, we performed GO and KEGG pathway enrichment analyses on these 7 candidate genes. GO enrichment analysis revealed that the candidate genes were mainly associated with chromosome- and cytoskeleton-related cellular components, including spindle pole (GO:0000922), mitotic spindle (GO:0072686), chromosome, centromeric region (GO:0000775), and cohesin complex (GO:0008278). Molecular function analysis identified enrichment in regulatory activities including NADPH binding (GO:0070402) and nuclear androgen receptor binding (GO:0050681) (Fig 3A). Exploratory KEGG pathway analysis identified nominal enrichment patterns involving autophagy-related pathways, AMPK signaling, FoxO signaling, and other stress-response-associated pathways; however, these pathways did not remain statistically significant after multiple-testing correction (Fig 3B).

**Fig 3:**
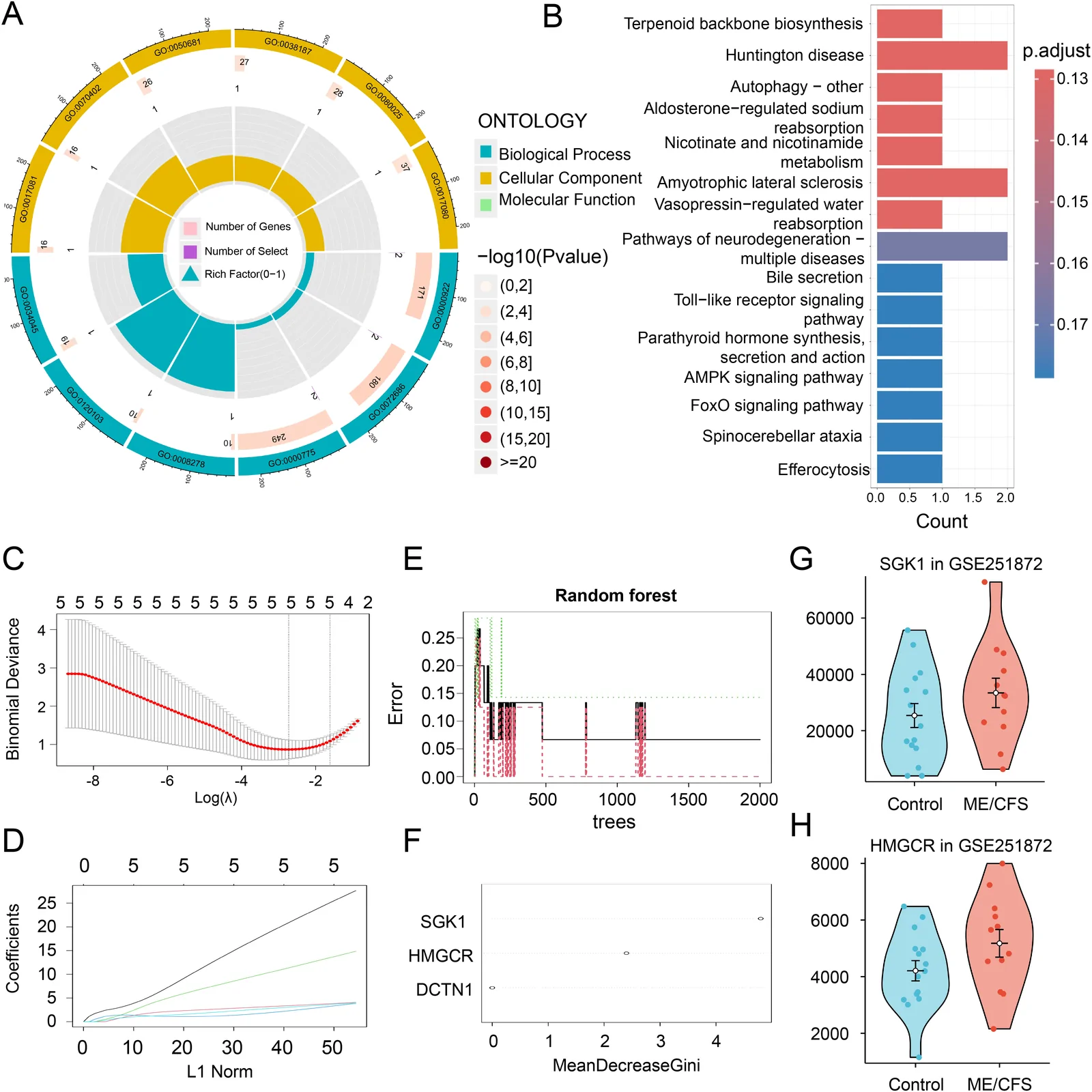
Functional enrichment, machine learning-based hub gene selection, and external evaluation of hub gene expression. A: GO circular plot: Biological Process (BP), Cellular Component (CC), Molecular Function (MF). B: Exploratory KEGG pathway enrichment analysis of candidate genes. Pathways shown were identified based on nominal enrichment patterns and did not remain statistically significant after multiple-testing correction. C: Lasso regression: Binomial deviance vs. Log(λ). D: Lasso coefficient profiles. E: Random Forest error vs. number of trees. F: MeanDecreaseGini ranking. G-H: External evaluation of SGK1 and HMGCR expression patterns in an independent PBMC RNA-seq cohort (GSE251872).

### 3.4 Machine learning identifies HMGCR and SGK1 as candidate hub genes

To further identify the most informative hub genes from the 7 candidates, we employed two machine learning algorithms: LASSO regression and RF. In the LASSO regression model, cross-validation determined the optimal lambda value minimizing binomial deviance, retaining genes with non-zero coefficients (Fig 3C, 3D). Concurrently, the Random Forest classifier ranked gene importance based on the Mean Decrease Gini index (Fig 3E, 3F). The intersection of results from both algorithms identified two core hub genes: HMGCR (3-hydroxy-3-methylglutaryl-CoA reductase) and SGK1 (serum/glucocorticoid regulated kinase 1). These genes showed strong classification performance within the analyzed dataset and were selected as candidate hub genes for subsequent investigation. To further assess the robustness of the identified hub genes, we examined SGK1 and HMGCR expression patterns in an independent PBMC RNA-seq cohort (GSE251872). Consistent with the discovery cohort, SGK1 showed an increased expression tendency in ME/CFS samples compared with healthy controls, although the difference did not reach statistical significance. A similar expression trend was also observed for HMGCR (Fig 3G, 3H).

### 3.5 Single-gene stratified GSEA identifies senescence-associated pathway signatures of HMGCR and SGK1

To investigate biological pathways associated with the expression patterns of HMGCR and SGK1, we performed single-gene stratified GSEA. Samples from the GSE14577 cohort were stratified into high- and low-expression groups according to the median expression level of each hub gene, followed by pathway enrichment analysis based on Hallmark and KEGG gene sets from the MSigDB. Compared with the HMGCR-low group, the HMGCR-high group exhibited significant enrichment of the “Cellular Senescence” hallmark pathway (adjusted P = 0.00192; Fig 4A). Additional pathways associated with higher HMGCR expression included VEGF signaling, NET formation, cell cycle regulation, and steroid biosynthesis (Fig 4B).

**Fig 4:**
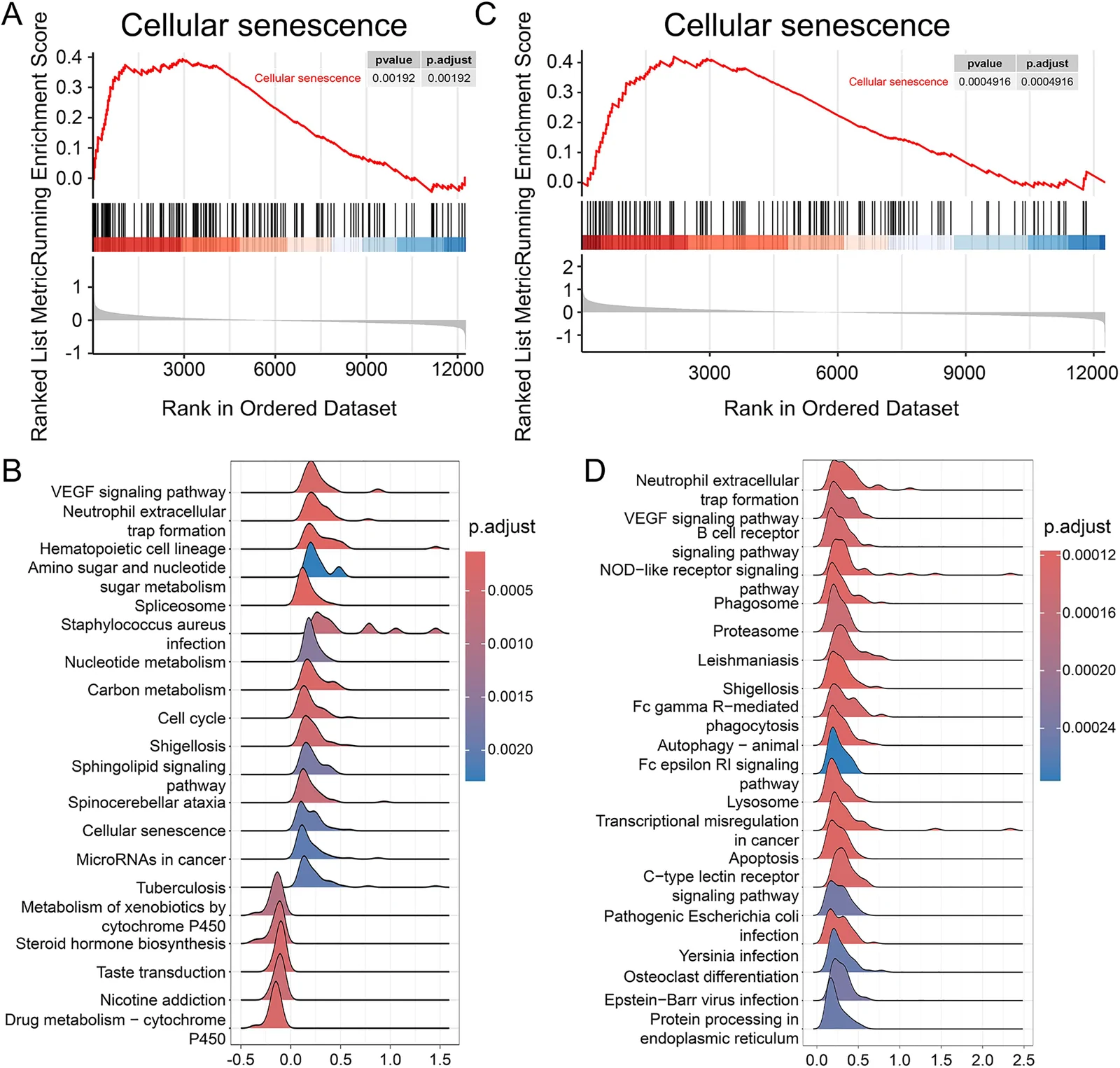
Single-gene stratified GSEA reveals senescence-associated pathway signatures of HMGCR and SGK1. A: HMGCR-associated “Cellular senescence” GSEA (p.adjust = 0.00192). B: Top pathways enriched by HMGCR: VEGF signaling, Neutrophil NETs, Cell cycle, Steroid biosynthesis. C: SGK1-associated “Cellular senescence” GSEA (*P*.adjust = 0.00049). D: Top pathways enriched by SGK1: FcγR-mediated phagocytosis, Autophagy, Lysosome, Apoptosis.

Similarly, the SGK1-high group showed significant enrichment of the “Cellular Senescence” hallmark pathway compared with the SGK1-low group (adjusted P = 0.00049; Figure 4C). Other pathways associated with higher SGK1 expression included FcγR-mediated phagocytosis, autophagy, lysosome function, and apoptosis-related processes (Figure 4D).

### 3.6 Single-cell transcriptomics characterizes cell-type-specific SGK1 expression patterns in ME/CFS and aging-associated immune states

To further characterize the cellular distribution of core genes and explore their relationship with aging-related immune alterations, we performed an exploratory integrated analysis using representative samples from two independent scRNA-seq datasets: GSE214283, derived from ME/CFS patients and healthy controls, and GSE157007, derived from aging individuals. Following strict quality control, UMAP dimensionality reduction identified major cell clusters, including monocytes, neutrophils, T cells, dendritic cells, and endothelial cells (Fig 5A). Mapping the expression of core genes onto the UMAP space revealed that SGK1 expression showed an increased tendency in ME/CFS and aging-associated samples compared with healthy controls (Fig 5B; S2A Fig). Notably, this upregulation was predominantly localized to monocyte and neutrophil populations. Furthermore, the expression pattern of SGK1 in ME/CFS patients closely resembled that observed in the aging cohort. In contrast to SGK1, HMGCR exhibited a broader and more diffuse expression pattern across different cell populations, without preferential enrichment in specific immune cell subsets. This distinct expression pattern suggests that HMGCR may represent a more broadly distributed metabolic-associated signature, whereas SGK1 shows a more cell-type-specific association with myeloid cell alterations in ME/CFS.

**Fig 5:**
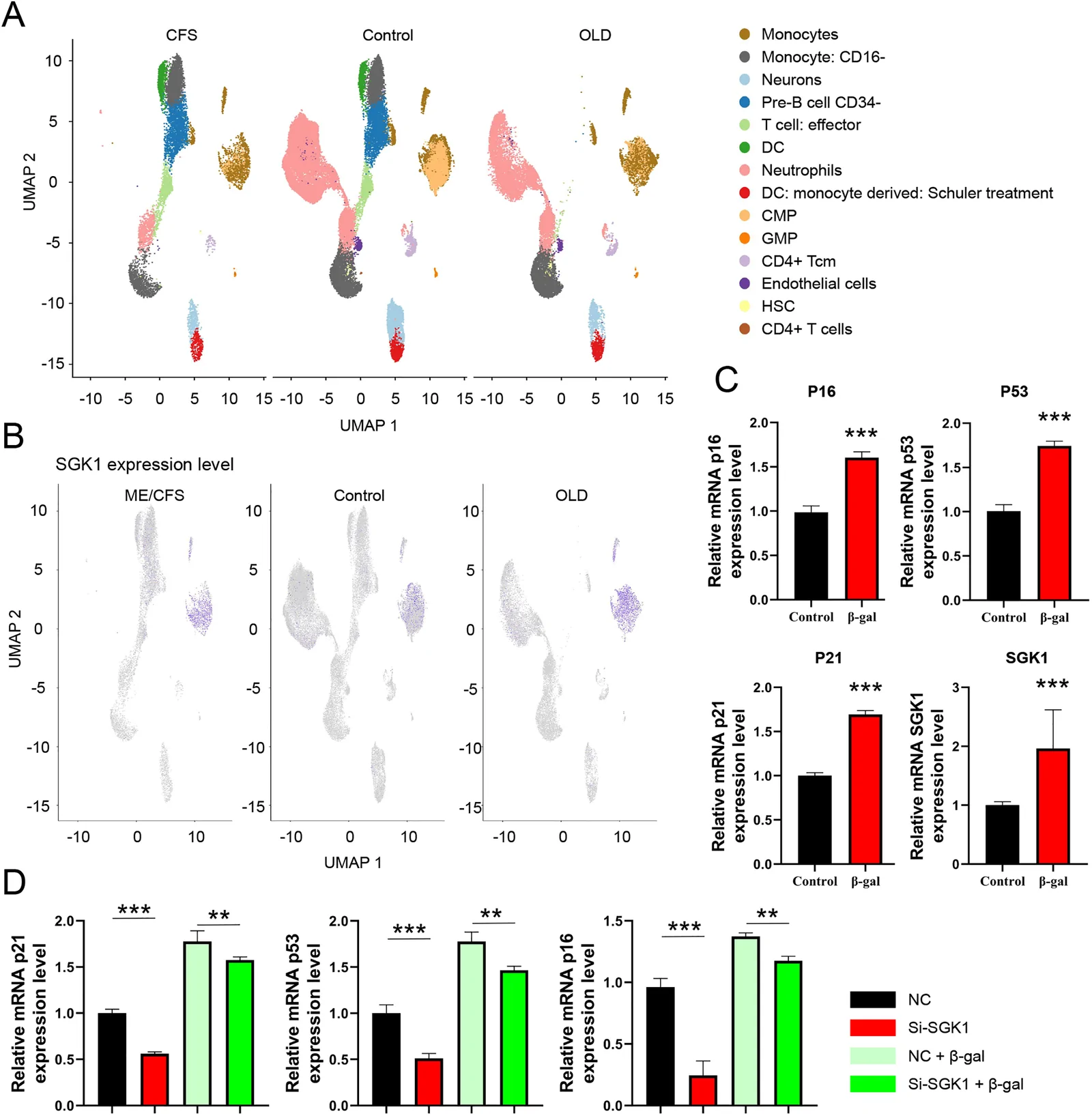
Single-cell transcriptomics and experimental analysis reveal the association of SGK1 with senescence-related alterations in ME/CFS. A: UMAP plot showing cell populations from an integrated analysis of 12 representative samples, including three ME/CFS samples and three healthy controls from GSE214283 and three healthy controls and three aging samples from GSE157007. B: SGK1 expression mapped on UMAP. SGK1 expression was predominantly observed in monocyte and neutrophil clusters in ME/CFS-associated samples. C: β-gal-induced senescence significantly upregulates mRNA levels of P16, P53, P21, and SGK1 (*P* < 0.001). D: Si-SGK1 significantly reduces P21, P53, and P16 mRNA levels (n = 3).

### 3.7 In vitro assessment of SGK1-associated senescence-related changes

To further explore the potential contribution of SGK1 to senescence-associated molecular changes, we established an in vitro cellular senescence model by treating THP-1 human monocytic cells with D-galactose (β-gal). In the senescence induction assay, D-gal treatment successfully induced a senescent phenotype, characterized by significant upregulation of classic senescence markers (p16, p53, p21), accompanied by a synchronous increase in SGK1 mRNA levels (*P* < 0.001; Fig 5C), indicating an association between SGK1 expression and the induced senescent phenotype. To further explore the potential involvement of SGK1 in senescence-associated molecular changes, we performed knockdown experiments using specific siRNA (si-SGK1). Transfection with si-SGK1 effectively suppressed SGK1 expression (S2B Fig). Moreover, silencing SGK1 attenuated several senescence-associated molecular changes, leading to a marked reduction in the mRNA levels of p21, p53, and p16 compared to the scrambled control group (*P* < 0.001; Fig 5D).

## 4. Discussion

The present study employed an integrative transcriptomic and experimental strategy to identify SGK1 as a candidate molecule associated with myeloid cell senescence-related alterations in ME/CFS. Through integrated analysis of bulk transcriptomic data, WGCNA, aging-related gene databases, and machine learning algorithms, SGK1 was identified as a core hub gene. Single-cell transcriptomic analysis revealed that SGK1 expression was enriched in myeloid cells from ME/CFS patients. In vitro experiments demonstrated that SGK1 expression was upregulated in a D-galactose-induced senescence model, and SGK1 knockdown reduced the mRNA expression levels of p16, p21, and p53. These findings suggest that SGK1 may be involved in the regulation of myeloid cell senescence, offering a mechanistic basis for understanding myeloid cell-mediated immune dysregulation in ME/CFS.

The datasets analyzed in this study were originally generated for investigating transcriptomic alterations associated with ME/CFS. While previous studies based on these datasets mainly focused on identifying differentially expressed genes and immune-related alterations, our study applied an aging-centered integrative analytical strategy to explore the potential relationship between molecular aging signatures and ME/CFS pathobiology. Therefore, our findings complement rather than replicate the conclusions of the original studies. SGK1 is a serine/threonine kinase regulated by various stress signals [18–20]. Previous studies have shown that SGK1 can modulate NF-κB activity and interact with the mTOR pathway [21,22]. NF-κB is a key transcriptional regulator of the SASP, while mTOR participates in its translational control [23,24]. These observations provide a potential mechanistic link between SGK1 and cellular senescence, although direct evidence in the context of ME/CFS is lacking. ssGSEA in the present study showed that SGK1 expression was associated with pathways related to autophagy and lysosomal function, processes known to be involved in senescence regulation. Based on these findings, it is plausible that SGK1 may be involved in myeloid cell senescence-associated processes through multiple pathways, but this hypothesis requires direct experimental validation. The enrichment of SGK1 in myeloid cells suggests that monocytes and related myeloid populations may represent one immune compartment associated with aging-related alterations in ME/CFS. However, this observation should be interpreted cautiously because the single-cell analysis performed in the present study was exploratory and based on representative samples rather than a population-level validation cohort. Therefore, whether SGK1-associated myeloid alterations represent a general feature of ME/CFS or occur only in specific patient subgroups remains to be determined. In addition, this finding does not exclude the contribution of other immune cell populations. Previous studies have reported immunosenescence-associated changes in lymphoid populations, including T cells and natural killer (NK) cells, indicating that immune aging in ME/CFS may involve multiple cellular mechanisms. The current study focused on myeloid cells because SGK1 showed preferential expression patterns in monocyte and neutrophil populations within the analyzed single-cell datasets. Whether SGK1-associated alterations in myeloid cells represent an independent contributor to immune aging or reflect downstream consequences of broader immune dysregulation remains unclear. Future studies integrating larger single-cell cohorts with multiple immune cell populations and detailed clinical annotation will be required to determine the relative contribution and interaction between myeloid and lymphoid immunosenescence in ME/CFS [3,25].

The present findings should be interpreted in the context of recent advances in ME/CFS research. Xiong and colleagues employed an AI-driven multi-omics approach to construct a “microbiome-immune-metabolome” axis interaction map in ME/CFS and identified activation of mucosal-associated invariant T cells and γδ T cells as key immunological features [10]. The present study, focusing on myeloid cell senescence, offers a complementary perspective on these systemic alterations, although the relationship between myeloid cell senescence and the observed immune activation requires further investigation. Natelson and colleagues documented cardiovascular abnormalities and metabolic disturbances in ME/CFS patients, which may be related to chronic inflammation [5]. Whether myeloid cell senescence contributes to such chronic inflammation remains to be determined. HMGCR was also identified as a core hub gene by machine learning algorithms. However, unlike SGK1, HMGCR did not demonstrate preferential enrichment within specific immune cell populations in the single-cell analysis. This broader expression pattern may indicate that HMGCR reflects a systemic metabolic-related alteration rather than a myeloid cell-specific senescence-associated signature. Therefore, while both genes represent ME/CFS-associated aging-related signatures, SGK1 may have a closer relationship with myeloid cell senescence-related alterations, which is further supported by the in vitro validation experiments. Adam reported a proposed blood test for ME/CFS based on single-cell Raman spectroscopy, which has garnered attention despite limited mechanistic understanding [12]. The identification of SGK1 in the present study may provide insights into molecular mechanisms underlying ME/CFS-related immune alterations and warrants further investigation as a potential molecular signature.

Several limitations of this study should be acknowledged. First, although external evaluation using the independent PBMC RNA-seq cohort GSE251872 showed a consistent expression tendency of SGK1, the bioinformatic analyses were primarily based on publicly available datasets with relatively limited sample sizes. Although GSE14577 provided a clinically characterized discovery cohort, the sample size was small and consisted exclusively of male participants, which may limit generalizability to the broader ME/CFS population, where females represent a substantial proportion of affected individuals. Therefore, validation in larger, sex-balanced cohorts with detailed clinical annotation is required. Second, although SGK1 showed consistent expression patterns across the discovery cohort and an independent ME/CFS PBMC RNA-seq dataset, the present study was not designed to establish SGK1 as a disease-specific diagnostic biomarker. Stress-responsive and inflammatory genes may exhibit altered expression across multiple chronic inflammatory or immune-mediated conditions. Therefore, future studies comparing SGK1 expression across ME/CFS, Long COVID, fibromyalgia, autoimmune diseases, and other inflammatory disorders will be necessary to determine whether SGK1 represents a ME/CFS-specific molecular signature or a broader marker associated with immune aging and inflammatory stress. Third, while the THP-1 cell line provides a useful model for investigating senescence-related changes, it may not fully recapitulate the behavior of primary monocytes from ME/CFS patients. The D-galactose-induced senescence model represents a pharmacological induction system that may differ from naturally occurring senescence processes in vivo. Future studies should validate SGK1 expression and senescence-associated phenotypes in primary monocytes isolated from ME/CFS patients. Fourth, although our transcriptomic analyses, single-cell characterization, and in vitro experiments support an association between SGK1 and senescence-related alterations, the current study does not establish SGK1 as a definitive causal regulator of myeloid cell senescence in ME/CFS. The molecular mechanisms potentially involving NF-κB, mTOR signaling, SASP factors, and autophagy pathways require further investigation. Fifth, the present study lacks in vivo evidence. Future studies using appropriate animal models, SGK1 genetic manipulation, or pharmacological intervention approaches will be necessary to further clarify the functional significance of SGK1 in ME/CFS-associated senescence-related processes.

Future studies should address several directions. Validation of SGK1 expression and its association with clinical phenotypes in larger, multi-center ME/CFS cohorts is warranted. Primary monocytes from ME/CFS patients should be examined directly for senescence-associated phenotypes and SGK1 expression levels. Mechanistic studies employing SGK1 genetic manipulation combined with systematic molecular approaches are needed to elucidate the pathways through which SGK1 regulates myeloid cell senescence. The therapeutic potential of SGK1 inhibition should be evaluated in animal models of fatigue or related phenotypes. Furthermore, the relationship between SGK1-associated myeloid senescence-related alterations and other ME/CFS-associated changes warrants further investigation. In conclusion, the present study introduces myeloid cell senescence as a potential molecular perspective for understanding immune dysregulation in ME/CFS and identifies SGK1 as a candidate molecule associated with senescence-related alterations. These findings provide a basis for further investigation, while additional studies are required to establish the clinical relevance and causal mechanisms of SGK1 in ME/CFS.

## Supporting information

S1 Fig Differential expression patterns between hub gene expression groups. (A) Differentially expressed genes between SGK1-high and SGK1-low groups. (B) Differentially expressed genes between HMGCR-high and HMGCR-low groups.
(TIF)

S2 Fig Donor-level characterization of SGK1 expression in monocytes and validation of SGK1 knockdown efficiency. (A) Donor-level characterization of SGK1 expression in monocyte populations derived from integrated single-cell RNA sequencing datasets. Expression values represent the average normalized SGK1 expression across monocytes from each donor. Each dot represents an individual donor from the Control, ME/CFS, or aging (OLD) groups. (B) Validation of SGK1 knockdown efficiency in THP-1 cells. Relative SGK1 mRNA expression was determined following transfection with control siRNA (NC) or SGK1-specific siRNA (Si-SGK1).
(TIF)

S1 Table Differentially expressed genes between ME/CFS patients and healthy controls in the GSE14577 dataset. (XLSX)

S1 Code R code for the integrative transcriptomic analysis workflow. (DOCX)
