# Supplementary material for: Identification of SGK1 as a candidate regulator associated with myeloid cell senescence in ME/CFS: An integrative transcriptomic and experimental study
Source: PLoS One. 2026 Oct 16;21(10):e0359082. doi: 10.1371/journal.pone.0359082 (PMC13674702; doi:10.1371/journal.pone.0359082)
Supplement: Supplementary file 1 [file pone.0359082.s004.docx]

############################################################

\# ME/CFS transcriptomic analysis workflow

\# Discovery cohort: GSE14577

\# Differential expression, aging-related gene screening,

\# WGCNA module analysis, machine learning selection,

\# pathway enrichment and validation

############################################################

library(tidyverse)

library(limma)

library(WGCNA)

library(clusterProfiler)

library(org.Hs.eg.db)

library(enrichplot)

library(glmnet)

library(randomForest)

library(pROC)

library(pheatmap)

options(stringsAsFactors = FALSE)

############################################################

\# 1. Expression matrix preprocessing

############################################################

exp <- read.table(

"GSE14577-GPL96_series_matrix.txt",

header = TRUE,

sep = "\t",

comment.char = "!"

)

gpl <- read.table(

"GPL96-57554.txt",

header = TRUE,

sep = "\t",

comment.char = "#"

)

probe_gene <- data.frame(

ID_REF = gpl$ID,

Gene_Symbol = gpl$Gene.Symbol

)

probe_gene$Gene_Symbol <- sapply(

probe_gene$Gene_Symbol,

function(x) strsplit(x,"///")[[1]][1]

)

exp <- merge(exp, probe_gene, by="ID_REF")

exp <- exp[!is.na(exp$Gene_Symbol) & exp$Gene_Symbol!="",]

expr <- avereps(

exp[,!colnames(exp) %in% c("ID_REF","Gene_Symbol")],

ID=exp$Gene_Symbol

)

expr <- as.matrix(expr)

############################################################

\# 2. Differential expression analysis

############################################################

group <- factor(c(

rep("Control",7),

rep("ME_CFS",8)

))

design <- model.matrix(~0+group)

colnames(design) <- levels(group)

fit <- lmFit(expr,design)

contrast <- makeContrasts(

ME_CFS-Control,

levels=design

)

fit <- contrasts.fit(

fit,

contrast

)

fit <- eBayes(fit)

deg <- topTable(

fit,

number=Inf,

adjust.method="fdr"

)

deg_sig <- deg[

abs(deg$logFC)>0.5 &

deg$adj.P.Val<0.05,

]

write.csv(

deg,

"DEG_all.csv"

)

write.csv(

deg_sig,

"DEG_significant.csv"

)

############################################################

\# 3. Aging-related gene intersection

############################################################

ARG <- read.table(

"Human_Aging_Related_Genes.txt",

header=FALSE

)

ARG <- unique(ARG[,1])

candidate_genes <- intersect(

rownames(deg_sig),

ARG

)

write.table(

candidate_genes,

"ARG_DEG_overlap.txt",

quote=FALSE,

row.names=FALSE,

col.names=FALSE

)

############################################################

\# 4. WGCNA

############################################################

datExpr <- t(expr)

goodSamplesGenes(

datExpr,

verbose=3

)

powers <- 1:20

sft <- pickSoftThreshold(

datExpr,

powerVector=powers

)

softPower <- sft$powerEstimate

net <- blockwiseModules(

datExpr,

power=softPower,

TOMType="unsigned",

minModuleSize=30,

mergeCutHeight=0.25

)

moduleGenes <- split(

rownames(net$colors),

net$colors

)

write.table(

moduleGenes,

"WGCNA_modules.txt",

quote=FALSE

)

############################################################

\# 5. Module trait association

############################################################

MEs <- orderMEs(

net$MEs

)

trait <- data.frame(

ME_CFS=as.numeric(group=="ME_CFS")

)

moduleTraitCor <- cor(

MEs,

trait,

use="p"

)

moduleTraitP <- corPvalueStudent(

moduleTraitCor,

nrow(datExpr)

)

write.csv(

moduleTraitCor,

"Module_trait_cor.csv"

)

############################################################

\# 6. Candidate gene selection

############################################################

module_candidates <- unique(

unlist(

moduleGenes[

names(moduleGenes) %in%

c("purple","darkgreen")

]

)

)

hub_genes <- intersect(

candidate_genes,

module_candidates

)

write.table(

hub_genes,

"Hub_candidates.txt",

quote=FALSE,

row.names=FALSE,

col.names=FALSE

)

############################################################

\# 7. LASSO selection

############################################################

x <- t(expr[hub_genes,])

y <- ifelse(

group=="ME_CFS",

1,

0

)

lasso <- cv.glmnet(

x,

y,

family="binomial",

alpha=1

)

coef_lasso <- coef(

lasso,

s="lambda.min"

)

selected <- rownames(coef_lasso)[

coef_lasso[,1]!=0

]

selected <- selected[selected!="(Intercept)"]

write.table(

selected,

"LASSO_selected_genes.txt",

quote=FALSE,

row.names=FALSE,

col.names=FALSE

)

############################################################

\# 8. Random forest validation

############################################################

rf_data <- data.frame(

expr[selected,],

Group=group

)

rf <- randomForest(

Group~.,

data=rf_data,

importance=TRUE

)

importance(rf)

############################################################

\# 9. Functional enrichment

############################################################

gene_id <- bitr(

hub_genes,

fromType="SYMBOL",

toType="ENTREZID",

OrgDb=org.Hs.eg.db

)

ego <- enrichGO(

gene_id$ENTREZID,

OrgDb=org.Hs.eg.db,

ont="ALL"

)

ekegg <- enrichKEGG(

gene_id$ENTREZID,

organism="hsa"

)

write.csv(

as.data.frame(ego),

"GO_enrichment.csv"

)

write.csv(

as.data.frame(ekegg),

"KEGG_enrichment.csv"

)

############################################################

\# 10. External validation

############################################################

validation_expr <- read.table(

"GSE251872_expression.txt",

header=TRUE,

sep="\t",

row.names=1

)

validation_hub <- validation_expr[

intersect(

selected,

rownames(validation_expr)

),

]

write.csv(

validation_hub,

"Validation_hub_expression.csv"

)

############################################################

\# End of workflow

############################################################

.libPaths(c("/R/seurat4", .libPaths()))

library(Seurat)

library(ggplot2)

library(tidyverse)

library(patchwork)

library(stringr)

library(RColorBrewer)

library(harmony)

library(SingleR)

library(celldex)

CT7 <- Read10X("./CT7/")[[1]]

CT8 <- Read10X("./CT8/")[[1]]

CT9 <- Read10X("./CT9/")[[1]]

OLD1 <- Read10X("./OLD1/")[[1]]

OLD2 <- Read10X("./OLD2/")[[1]]

OLD3 <- Read10X("./OLD3/")[[1]]

CT4 <- Read10X_h5(

"./CT4/GSM6603124_COR-9546-D1_filtered_feature_bc_matrix.h5"

)

CT5 <- Read10X_h5(

"./CT5/GSM6603116_COR-1126-D1_filtered_feature_bc_matrix.h5"

)

CT6 <- Read10X_h5(

"./CT6/GSM6603210_COR-7717-D1_filtered_feature_bc_matrix.h5"

)

CFS1 <- Read10X_h5(

"./CFS1/GSM6603110_COR-7349-D1_filtered_feature_bc_matrix.h5"

)

CFS2 <- Read10X_h5(

"./CFS2/GSM6603113_COR-6215-D1_filtered_feature_bc_matrix.h5"

)

CFS3 <- Read10X_h5(

"./CFS3/GSM6603115_COR-3717-D1_filtered_feature_bc_matrix.h5"

)

CT4 <- CreateSeuratObject(CT4, project="CT4")

CT5 <- CreateSeuratObject(CT5, project="CT5")

CT6 <- CreateSeuratObject(CT6, project="CT6")

CT7 <- CreateSeuratObject(CT7, project="CT7")

CT8 <- CreateSeuratObject(CT8, project="CT8")

CT9 <- CreateSeuratObject(CT9, project="CT9")

CFS1 <- CreateSeuratObject(CFS1, project="CFS1")

CFS2 <- CreateSeuratObject(CFS2, project="CFS2")

CFS3 <- CreateSeuratObject(CFS3, project="CFS3")

OLD1 <- CreateSeuratObject(OLD1, project="OLD1")

OLD2 <- CreateSeuratObject(OLD2, project="OLD2")

OLD3 <- CreateSeuratObject(OLD3, project="OLD3")

scRNA <- merge(

CT4,

y=c(

CT5,

CT6,

CT7,

CT8,

CT9,

CFS1,

CFS2,

CFS3,

OLD1,

OLD2,

OLD3

)

)

scRNA$ tissue_type <- str_remove(

scRNA$orig.ident,

"[0-9]"

)

scRNA[["percent.mt"]] <-

PercentageFeatureSet(

scRNA,

pattern="^MT-"

)

HB.genes <- c(

"HBA1",

"HBA2",

"HBB",

"HBD",

"HBE1",

"HBG1",

"HBG2",

"HBM",

"HBQ1",

"HBZ"

)

HB.genes <- rownames(scRNA)[

match(

HB.genes,

rownames(scRNA)

)

]

HB.genes <- HB.genes[

!is.na(HB.genes)

]

scRNA[["percent.HB"]] <-

PercentageFeatureSet(

scRNA,

features=HB.genes

)

scRNA <- subset(

scRNA,

subset=

nFeature_RNA > 200 &

nFeature_RNA < 3000 &

percent.mt < 17.5 &

nCount_RNA < 10000

)

scRNA <- NormalizeData(

scRNA,

normalization.method="LogNormalize",

scale.factor=10000

)

scRNA <- FindVariableFeatures(

scRNA,

selection.method="vst",

nfeatures=2000

)

scRNA <- ScaleData(

scRNA,

features=VariableFeatures(scRNA)

)

scRNA <- RunPCA(

scRNA,

features=VariableFeatures(scRNA)

)

scRNA <- RunHarmony(

scRNA,

group.by.vars="orig.ident"

)

scRNA <- FindNeighbors(

scRNA,

dims=1:16,

reduction="harmony"

)

scRNA <- FindClusters(

scRNA,

resolution=0.8

)

scRNA <- RunUMAP(

scRNA,

dims=1:16,

reduction="harmony"

)

library(SingleR)

library(celldex)

load("HumanPrimaryCellAtlasData.Rdata")

load("bpe.se.Rdata")

load("ImmGenData.Rdata")

load("NovershternHematopoieticData.Rdata")

refdata <- list(

hpca.se,

bpe.se,

img.se,

nsh.se

)

seurat_version <- packageVersion("Seurat")

if (seurat_version >= "5.0.0") {

if (length(Layers(scRNA)) > 1 &&

any(grepl("^data\\.", Layers(scRNA)))) {

scRNA_temp <- JoinLayers(scRNA)

testdata <- GetAssayData(

scRNA_temp,

layer="data"

)

} else {

testdata <- LayerData(

scRNA,

layer="data"

)

}

} else {

testdata <- GetAssayData(

scRNA,

slot="data"

)

}

cellpred <- SingleR(

test=testdata,

ref=refdata,

labels=list(

hpca.se$label.fine,

bpe.se$label.fine,

img.se$label.main,

nsh.se$label.main

),

clusters=scRNA$seurat_clusters,

assay.type.test="logcounts",

assay.type.ref="logcounts"

)

if (exists("scRNA_temp")) {

rm(scRNA_temp)

}

celltype <- data.frame(

ClusterID=rownames(cellpred),

celltype=cellpred$labels

)

scRNA$celltype <- "NA"

for(i in 1:nrow(celltype)){

scRNA$celltype[

scRNA$seurat_clusters == celltype$ClusterID[i]

] <- celltype$celltype[i]

}

Idents(scRNA) <- scRNA$celltype

DimPlot(

scRNA,

reduction="umap",

split.by="tissue_type"

)

genes <- c(

"SGK1",

"HMGCR"

)

FeaturePlot(

scRNA,

reduction="umap",

features=genes,

split.by="tissue_type"

)

DotPlot(

scRNA,

features=genes

)

VlnPlot(

scRNA,

features=genes,

pt.size=0,

log=TRUE

)

saveRDS(

scRNA,

file="scRNA_anno.RDS"

)

scRNA <- readRDS(

"scRNA_anno.RDS"

)

mono_cells <- subset(

scRNA,

subset=

celltype %in% c(

"Monocytes"

)

)

mono_exp <- FetchData(

mono_cells,

vars=c(

"SGK1",

"HMGCR",

"orig.ident",

"tissue_type"

)

)

donor_exp <- mono_exp %>%

group_by(

orig.ident,

tissue_type

) %>%

summarise(

SGK1_mean=mean(SGK1),

HMGCR_mean=mean(HMGCR),

n_cells=n(),

.groups="drop"

)

p_SGK1 <- ggplot(

donor_exp,

aes(

x=tissue_type,

y=SGK1_mean,

fill=tissue_type

)

)+

geom_boxplot(

width=0.55,

alpha=0.3,

outlier.shape=NA

)+

geom_jitter(

width=0.12,

size=3

)+

theme_classic()+

labs(

x=NULL,

y="Mean SGK1 expression"

)+

theme(

legend.position="none"

)

p_HMGCR <- ggplot(

donor_exp,

aes(

x=tissue_type,

y=HMGCR_mean,

fill=tissue_type

)

)+

geom_boxplot(

width=0.55,

alpha=0.3,

outlier.shape=NA

)+

geom_jitter(

width=0.12,

size=3

)+

theme_classic()+

labs(

x=NULL,

y="Mean HMGCR expression"

)+

theme(

legend.position="none"

)

p_final <- p_SGK1 + p_HMGCR

ggsave(

"Supplementary_Figure_Donor_level_SGK1_HMGCR.pdf",

p_final,

width=8,

height=4

)

write.csv(

donor_exp,

"Donor_level_SGK1_HMGCR_expression.csv",

row.names=FALSE

)
